# Efficiency Measurement and Heterogeneity Analysis of Chinese Cultural and Creative Industries: Based on Three-Stage Data Envelopment Analysis Modified by Stochastic Frontier Analysis

**DOI:** 10.3389/fpsyg.2021.823499

**Published:** 2022-01-27

**Authors:** Mingxing Li, Hongzheng Sun, Fredrick Oteng Agyeman, Jialu Su, Weijun Hu

**Affiliations:** ^1^School of Management, Jiangsu University, Zhenjiang, China; ^2^Department of Cultural Heritage and Museum Studies, School of Archaeology, Jilin University, Changchun, China

**Keywords:** cultural and creative industries, three-stage DEA model, stochastic frontier analysis, listed enterprises, technical efficiency, operation efficiency

## Abstract

Industry sustainability plays a vital role in shaping the environment for cultural and creative business development. However, considering the influence of the external environment and random factors on the technical efficiency (T.E.) of cultural and creative industries with the inherent defects of the traditional data envelopment analysis (DEA) model; this manuscript analyzed the operating efficiency of 56 cultural and creative enterprises using the three-stage DEA model from 2012 to 2018. An analysis of the results shows that differences in efficiency exist between stage one and stage three DEA. Furthermore, the environmental elements and statistical noise measured by the stochastic frontier analysis (SFA) in stage two reveal positive and negative influences on the creative cultural enterprises at different stages. As a result, the overall efficiency of the listed cultural and creative industries was revealed to be low in China. Finally, this study suggested effective countermeasures and recommendations for better-operating efficiency systems for cultural and creative enterprises.

## Introduction

The Cultural and Creative Industries (CCIs) in the 21st century have attracted global attention with their unique qualities and astonishing growth (BOP, 2010; Puchta et al., 2010; Johnson et al., 2019; Mazilu et al., 2020). The cultural industries with creativity as the crux of the world occupy a pivotal position connected to gross domestic products (GDP) contribution, production scale, and international trade cooperation (Skavronska, 2017; Li, 2018; Wang and Liu, 2021). Growing studies on CCIs reveal the enormous contribution to the nation’s economy in particular and the global economic development in general (Jones et al., 2004; Van Der Pol, 2007; De Beukelaer, 2014; Correa-Quezada et al., 2018). Considering the role of similar industries, the CCIs have significant characteristics such as low resource consumption, low environmental pollution, intense creativity, and high added worth (Kačerauskas, 2012). In addition, CCIs provide an enormous number of jobs while accomplishing tenable development and high-technology interaction (Jeffcutt et al., 2000; Jeffcutt, 2007; Budnitsky and Jia, 2018; Prados-Peña and Del Barrio-García, 2021). Current statistics indicate that China represents a leading CCI in Asia and recorded € 50.32 billion in revenue resulting in 2.45% of her GDP and a growth of 6.4% progress of the economy in general (Li and Ju, 2020). In 2015, the Chinese CCIs grew 11%, translating to 2,723.5 billion CNY (Mélanie and Champagne, 2017). Referring to the National Bureau of Statistics Records in 2017, the added worth of China’s CCIs accounted for more than 4% of GDP, with a highly dynamic performance during the period. With the deepening of innovation and the unification of the “Internet plus Culture,” the cultural information transmission service industry has developed rapidly (Eiroa and Magallón, 2018; Altan and Sunay, 2020). Although cultural and creative enterprises play an essential role in the economic development of industrialized countries, they tend to be small and often fail due to various constraints and tensions (Landoni et al., 2020). A statistical report shows that while the CCI scale has expanded and revenues have increased (National Bureau of Statistics of China [NBSC], 2020), about 56 out of 132 cultural-related enterprises representing 42.42% were disclosed as negative growth in net profits in the 2016 annual report in China (Yongfen et al., 2020).

Thus, this present study is based on an important question: why are these companies reporting negative profit growth? Could it be that there are certain inefficiencies in their input and output variables at certain stages of their production? If so, these inefficiencies could put some constraints on the active development of China’s CCIs and need to be addressed.

The most attributable reason for this phenomenon is low input–output efficiency. The poor operational efficiency of these companies can cause inefficiencies throughout the industry, thus hampering the active development of the CCIs in China. The advancement of a creative economy is an established national policy for the sustainable growth of developed countries and an essential guide for developing and less developed countries (Daubaraitė and Startiene, 2017; Grushka, 2018; Asghar et al., 2021). The swift rise of the CCIs has aroused great attention from the academic community (Jones et al., 2015; Lazzeretti et al., 2017, 2018). It is, therefore, necessary to fully understand the growth of the industry, measure the technical efficiency (T.E.) of CCIs, and explore the influencing factors in their development process (Bayaraa et al., 2019). Hence, this study bridges the gap created in Chinese literature and uses the three-stage DEA model to ascertain the technical efficiency value of China’s Cultural and Creative Listed Companies and exclude the brunt of environmental elements and statistical noise on the evaluated results to make the analysis more objective. The study will further enrich the academic debate by contributing to the existing knowledge on CCIs.

## Literature Review

Several academic explorations have been conducted on the CCIs in different nations, regions, and cities (Pinheiro and Hauge, 2014; Shan, 2014; Tang, 2016). Many academicians have referred to the CCIs as Creative Industries (C.I.s), Cultural Product Industries, the Cultural Economy, and the Creative Economy (Galloway and Dunlop, 2007; Boggs, 2009; Grodach, 2013; Shehzad et al., 2020). However, the authors of this present study assert that all the names referring to the CCIs are the same.

Most scholars have explored the efficiency and influencing factors of profit and non-profit public sectors such as cultural services in recent years (Paulsen and Business, 2006; Amirkhanyan et al., 2008; Kallifatides and Neubeck, 2020; Yongfen et al., 2020). For example, Bishop and Brand (2003) found that higher public funding and public welfare activities negatively impacted the T.E. of museums in Southwest England. Marco-Serrano (2006) pointed out that the improvement in productivity of the CCIs needs a conducive environment and technology. An empirical study on whether social networks always promote entrepreneurial intentions indicated that three social network dimensions, network size, network heterogeneity, and top node properties, affect entrepreneurial intentions significantly (Xiao and Fan, 2014; Shah et al., 2021). The application of social network technology to culture and creative enterprises will boost the performance of CCIs. Also, the presence of cultural venues and facilities, the jobs and innovations linked to the creative industries, and the enabling conditions for culture and creativity diffusion: human capital, diversity, trust and openness, international accessibility and connectivity, can enhance the dynamism of cultural and creative industries (Bertoni et al., 2021). With the improvement of efficiency, the entry of more groups may bring about changes in the frontier structure of cultural production, which was found to have reduced the efficiency of the Spanish theater. Based on the stochastic frontier analysis (SFA), Wetzel (2010) found that the subsidies from the German government have a specific undesirable bearing on the T.E. of the German public theater resulting in a reduction in average T.E. and, thus, proposed that the government should focus on cost minimization incentives. Figueroa et al. (2018) studied Chile’s tourism sector by exploring cultural factors and their effects on tourist attractions’ operational efficiency. Several intellectuals have also deliberated on the significance of organizational innovation on CCIs. For example, Savino et al. (2017) used case-based analysis to examine how creative cultural firms implement innovation through a specific team of creators.

In China, the CCIs research is mostly conducted on the inter-provincial, regional, and enterprise perspectives using panel data to explore efficiency and its influencing factors in China. Jia-ting and Ting (2013) and Jalloh and Guevera (2017) stated that the standard of regional economic development, cultural market demand, social capital, and government policy support could enhance the efficiency of the CCIs. In contrast, many enterprises and the irrational cultural system inhibited the industry’s evolution. From the perspective of micro-enterprise, Roy (2009) and Lin et al. (2017) found that the proportion of state-owned shares, employees’ education level, and the relative marketing scale positively impacts business efficiency. Founded on the DEA method, Yu et al. (2014) looked into the different financing modes of listed corporations in the CCIs. Their results depicted an adverse correlation between corporate financing efficiency and intangible assets. The study suggested that enterprises should, therefore, reduce the inefficiency of financing inputs and avoid misuse of resources (Asghar et al., 2020). Zhao et al. (2015) constructed an SFA model founded on transcendental logarithmic production function with 70 creative cultural enterprises and measured the industrial T.E. from 2010 to 2014. Their findings show that enterprises’ T.E. value is generally low, and its changing trend shows slightly negative growth. Also, in adopting the transcendental logarithmic SFA method (Liu et al., 2017) used the panel data of regional animation enterprises to investigate the impacts of intellectual property rights on the T.E. of the CCIs from the three dimensions of intellectual property creation, application, and management. The outcome was that external intellectual property protection positively affects the T.E. of the CCI. Hu and Zhou (2018) and Ajaz et al. (2020) took the listed cultural companies in Shanghai and Shenzhen and explored the efficiency evaluation of listed cultural companies under the heterogeneity of DMUs. The current change in trends of enterprises’ performance has become a common phenomenon nowadays. A study on Chinese military-industrial enterprises within the ‘10 Major Military-industrial Groups’ emphasized a gradual increase in technical efficiency; however, the overall outcome was low (Mingxing et al., 2019; Mohsin et al., 2021).

In summary, the above-analyzed studies indicate research works that have used different methods to explore the CCIs and their influencing factors from different perspectives; there are very few empirical works conducted on the ultimate purpose of the subject matter. Most researchers use provincial and municipal regional panel data, lacking practical significance. Also, there are some limitations found in the research methods of the existing literature. Further, most studies employ the traditional DEA model that cannot effectively deal with the partial missing data. Again, many scholars do not consider the impact of environmental and random factors on the evaluated subject. The traditional SFA model also defines an aspect of the production frontier function that may lead to efficiency measurement bias. In contributing to knowledge and academia, this current study is founded on the panel data of 136 listed cultural and creative enterprises using data from 2012 to 2018. This study employs a three-stage DEA and modified SFA models to measure the cultural enterprises’ technical efficiency. Also, it effectively filtered out the deviations caused by environmental factors and statistical noise of the enterprises to conform to the operational standards, which yielded an accurate result for the study.

## Research Methods

The three-stage DEA model was initially proposed and applied by Fried et al. (2002). In stage one, the (Banker et al., 1984) (BCC) DEA method was used to analyze the input–output data to attain an initial evaluation of enterprise performance. However, this evaluation does not account for the brunt of environmental elements and statistical noise. Hence, in stage two, the authors of this study applied the stochastic frontier analysis (SFA) model to eliminate the brunt of environmental elements and statistical noise found in the traditional DEA model of the study. The SFA model made the analysis of the management inefficiency more accurately. The SFA model has been lengthily applied in many areas such as bank efficiency, regional innovation efficiency, resource management, and environmental efficiency; however, few scholars have used this method to investigate the efficiency of the CCIs (Ping and Yong, 2011; Chunfa and Xiangli, 2013; Jia-ting and Ting, 2013; Zeng et al., 2016). The research outcomes showed a change in inefficiency after filtering the brunt of environmental elements and statistical noise to efficiency.

In stage three, the input–output performances are adjusted to account for the environmental elements and statistical noise discovered in stage two. The re-assessment of enterprise efficiency in stage three offers a better managerial efficiency test since the environmental factors and white noise has been expunged. The creation and operation of the model are mainly made up of three phases:

### Stage One

The main principle of data envelopment analysis (DEA) is to use the initial input–output data of decision-making units (DMUs). The mathematical programming is used to ascertain the relative effective production frontier and to liken the relative efficiency between the DMUs by realizing the projection size on the DMUs to the production frontier (Wang et al., 2014; Chun et al., 2015; Yang et al., 2015). Thus, this manuscript uses the input-oriented Banker, Charnes, and Copper (BCC) model in stage one for the analysis. The model is founded on variable returns to scale (VRS) and is capable of analyzing the integrated T.E. and its decomposition of pure technical efficiency (PTE) and scale efficiency (S.E.), which is calculated as SE = TE/PTE. Since the traditional DEA model is very mature, its mathematical principles are well documented in many studies and will not be repeated. The initial efficiency values calculated in stage one are compared and analyzed in the subsequent steps. The slack variables of all inputs in phase one will be adopted as the dependent variables of stage two.

### Stage Two

By constructing the SFA regression model, the *m* input slack variables of *n* decision-making unit in stage one are used as the dependent variables of stage two, which are decomposed into the functions of three independent variables, including environmental elements, statistical noise, and management factors. Thus, the expression is as follows:


(1) $$S_{i\,j}=f^{\text{i}}\,\left(z_{j};\beta^{\text{i}}\right)+v_{i\,j}+\mu_{i\,j}$$

Where *S*_*ij*_ is expressed as the slack variable of the *j^th^* decision unit on the i^*th*^ input, that is, the disparity between the ideal input and the actual input, which is expressed as:


(2) $$s_{i\,j}=x_{i\,j}-X_{i}\,\lambda$$

*f*^i^(*Z*_*j*_β^i^) indicates the environmental elements on slack variables, generally, *f*^i^(*z*_*j*_;β^i^)=*z*_*j*_β,*Z*_*j*_ is the observed k-dimensional environment variables, β^i^ indicates the parameter to be evaluated for the environmental variable. *v*_*ij*_+μ_*ij*_ mixed error term, where, $v_{i\,j}\approx N\,\left(0,\sigma_{v}^{2}\right)$, is the normal distribution, reflecting white noise; $\mu_{i\,j}\approx N\,\left(0,\sigma_{\mu }^{2}\right)$ denotes a truncated non-negative normal distribution, which is usually implicit in obeying a semi-normal distribution (Greene, 1990), reflecting management inefficiency, and is independent and unrelated to each other. In defining $\gamma =\frac{\sigma_{\mu }^{2}}{\sigma_{\mu }^{2}+\sigma_{v}^{2}}$ thus, when γ tends to 1, it shows that the influence of management factors is enormous, and the maximum likelihood approximation is adopted. Conversely, when it tends to 0, it signifies that random factors’ influence is significant, and the least-squares estimation should be adopted. Using FRONTIER 4.1 to calculate the parameters β^*i*^,σ^2^ where, $\sigma^{2}=\sigma_{v}^{2}+\sigma_{\mu }^{2}$, the authors adjust the initial input data and restructured the formula as follows:


(3) xi⁢j*=xi⁢j+[max⁡(zj⁢βi)-zj⁢βi]+[max⁡(vi⁢j)-vi⁢j]

Where *i*=1,2,3,…,*m*;*j*=1,2,3,…*n*

xi⁢j* represents the adjusted input value, the first brackets adjust the impact of environmental elements, and the second bracket indicates filtering white noise. Separating the management inefficiency term is a crucial step in this stage. This manuscript uses the management inefficiency estimation formula (Dengyue, 2012).


(4) $$\text{Ê}\,\left(\mu_{\text{ij}}|\mu_{\text{ij}}+{\text{v}}_{\text{ij}}\right)=\frac{\lambda \,\sigma }{1+\lambda^{2}}\,\left[\frac{\varphi \,\left(\varepsilon_{\text{i}}\,\lambda /\sigma \right)}{\phi \,\left(\varepsilon_{\text{i}}\,\lambda /\sigma \right)}+\frac{\varepsilon_{\text{i}}\,\lambda }{\sigma }\right]$$

where, $\lambda =\frac{\sigma_{\mu }}{\sigma_{\upsilon }},\varepsilon_{i}=\upsilon_{i\,j}+\mu_{i\,j}$ are the joint error terms, φ, ϕ the density function, and the distribution function of the standard normal distribution, respectively. Based on this, the conditional estimate of the white noise is obtained as follows:


(5) $$\text{Ê}\,\left(\upsilon_{i\,j}|\mu_{i\,j}+v_{i\,j}\right)=s_{i\,j}-Z_{j}\,\beta^{i}-\text{Ê}\,\left(\mu_{i\,j}|\mu_{i\,j}+v_{i\,j}\right)$$

The primary purpose of stage two is to adjust all DMUs to the same environment level; even if all have similar experience conditions, the DMU with good conditions increases their input. Conversely, the DMU with less favorable conditions yields less in inputs.

### Stage Three

The new input value X_*ij*_, which filters out environmental elements and white noise, is brought into the traditional DEA model to calculate the relative efficiency value of each DMU. As a result, the measured results can better reflect the input–output level of the listed cultural and creative enterprises.

## Variable Selection and Data Description

The CCIs are composed of many micro-enterprises. This manuscript selects 56 listed enterprises whose primary business content is cultural creativity. The 56 cultural enterprises comprise 6 creative design service enterprises, 32 cultural publishing media enterprises, 8 cultural creative production enterprises, and 10 cultural leisure and entertainment enterprises. This study’s analysis period spans from 2012 to 2018.

### Input and Output Variable Selection

According to the classical and endogenous economic growth theories, capital and labor are chosen as the essential input factors based on the production functions and general standard (Doepke and Zilibotti, 2014; Petchko, 2018). The enterprise’s fixed assets measure the total capital investment input index; employees’ annual salary measures the labor input index. The output indicator is measured by its primary yearly total business income (Zhao et al., 2015). The input–output indicator’s data sources are all annual reports of the enterprise, which are accurate and reliable. The input–output variables should conform to the “homogeneity” hypothesis in the DEA model, assuming that one variable cannot increase as the other decreases. Table 1 outlines the variables used for this study, and Table 2 gives a descriptive statistic of the input–output variables. This manuscript employs the Pearson correlation test to authenticate the results, as displayed in Table 3. Table 3 shows that the correlation coefficient between each enterprise’s input–output variables passes the two-tailed test at the significant level of 1%. Thus, a significant positive correlation is consistent with the principle of “homogeneity” and suggests a reasonable selection of indicators.

**TABLE 1:** Variable description.

Category	Variables	Description
Input variable	Total capital investment	Capital investments are resources invested in an enterprise for the main purpose of fostering its business objectives. It may also relate to a firm’s procurement of capital or fixed assets, for instance, manufacturing machinery, which has been assumably productive for many years.
	Total cost of labor	The cost of labor is the totality of all salaries paid to workers, inclusive of the total of all worker entitlements and payroll taxes paid by an employer.
Output variable	Total business income	The amount of all money received by an individual/organization, including income from service or providing services, proceeds from sales, expenditures from pension plans, revenue from dividends, or other sources.

**TABLE 2:** Descriptive statistics of input/output variables from 2012 to 2018.

		Mean	Maximum	Minimum	Standard deviation
Inputs	Total capital investment	1064	10037	0.19	1657
	Total cost of labor	75	1230	0.039	136
Output	Total business income	3264	96055	136	7242

*Unit in million CNY.*

**TABLE 3:** Pearson correlation coefficient between input and output variables.

Correlations			

		Capital investment	Labor input
Output	Pearson correlation	0.170***	0.235***
	Sig. (two-tailed)	0.001	0.000
	*N*	392	392

*** indicates a significant correlation at the 1% level (both sides).

### Environmental Variable Selection

Considering the development characteristics of the CCIs, the manuscript analyzes the environmental elements that affect their T.E., from the facets of enterprise size (science and technology), ownership structure, talent factor, and government factors.

#### Enterprise Size (Science and Technology)

The enterprise size determines the issuance of corporate bonds, and the magnitude of the bond financing has a positive correlation with enterprise innovation performance (Wang et al., 2018; Uzochukwu et al., 2020). This manuscript selects the customer concentration of each enterprise as the proxy variable. Customers’ focus has a substantial adverse effect on an organization’s research and development. Notwithstanding, research and development are the basis for developing cultural and innovative enterprises, providing a constant stream of development ideas (Sundbo, 1997; Wu et al., 2017).

#### Ownership Structure

The concentration of ownership structure is selected as the explicit indicator of this study. The shareholding ratio of the enterprises shows the concentration of the top 10 ownership structures. Equity concentration is an environmental factor that a company cannot make significant alterations in a short period, and it has a significant positive correlation with corporate performance (Liping et al., 2006).

#### Talent Factor

Selecting the proportion of employees having a bachelor’s degree as a talent factor (Jia-ting and Ting, 2013) showed that high-quality creative talents have apparent effects on improving the T.E. of enterprises. This is because the generation and application of creativity hinge heavily on talents. Furthermore, affected by regional factors, high-quality creative skills are more abundant in developed regions, but underdeveloped areas often have difficulties introducing talents.

#### Government Factors

As a strategic emerging industry, the government has formulated many supportive policies to promote its healthy development from the facets of capital and talents. This manuscript’s government factor is the local government’s subsidy granted to the creative cultural enterprise. The proxy variable is the government subsidy of the enterprise’s annual operating income. The data for environmental factors was acquired from each enterprise’s annual reports. These four factors were analyzed by collinearity using the SPSS 24.0 software. The results are shown in Table 4.

**TABLE 4:** Collinear diagnosis.

Environmental variable	Tolerance	VIF	Model	Dimension	Eigenvalues	Conditional index
Customer concentration	0.990	1.010	1	1	3.637	1
Proportion of the top 10 shareholders	0.980	1.021		2	0.720	2.247
Bachelor degree or above	0.989	1.011		3	0.433	2.898
Government subsidy	0.986	1.014		4	0.185	4.434

From Table 4, the VIF is less than 5, and the conditional index is less than 10, suggesting no serious collinearity problem between environmental variables. Thus, the selection of the indicators for the study is appropriate.

## Empirical Analysis

### Empirical Results of Stage One Data Envelopment Analysis Model

**FIGURE 1:**
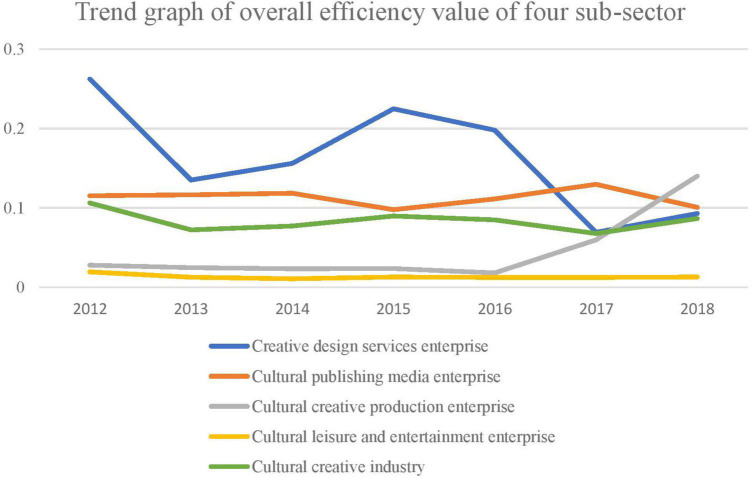
Adjustment of the top four enterprises and cultural creative industry’s overall efficiency value (2012–2018 average).

Using MaxDEA software to measure each enterprise’s annual efficiency values, the stage one DEA model outcomes are shown in Table 5. Under the brunt of environmental elements and statistical noise, the efficiency value of cultural and creative enterprises as a whole showed a dynamic trend, as shown in Figure 1. The year 2012 recorded the highest efficiency value of 0.1062. Contrarily, the efficiency values of the remaining 6 years dwindled, with their accompanying value lower than 0.1. Analysis derived from the sub-sectors perspective indicated that the efficiency of cultural leisure and entertainment enterprises was recorded lower than the overall efficiency. Likewise, the other three sub-sectors revealed a highly sluggish downward trend. The creative design service industry had the most considerable fluctuation in the efficiency value and growth rate. Before 2016, it was ahead of other subdivided industries; however, after 2016, it began to experience competition from the different markets, affecting growth and innovation diffusion (Badia et al., 2020). The cultural publishing media and cultural creative production enterprise’s performance was higher than the creative design service enterprise in 2017. Findings indicated in 2016 that the efficiency value of the cultural publishing media enterprises was relatively stable while cultural creative production enterprises dwindled. Notwithstanding, it had a huge turning point after 2016, having a rapid upward trend in the efficiency value. Furthermore, in 2018 it successfully surpassed the other three sub-sectors, reflecting the industry’s vast development space and potential.

**TABLE 5:** Technical efficiency values of creative culture enterprises in stage one.

Years		2012	2013	2014	2015	2016	2017	2018	Mean	Ranking

Enterprise										
Creative design service enterprise	300052	0.0481	0.0411	0.0481	0.0668	0.0814	0.0283	0.0340	0.0497	**18**
	300264	0.1327	0.1247	0.1038	0.1295	0.1374	0.0826	0.1182	0.1184	**12**
	300182	0.0811	0.0785	0.1195	0.3599	0.0230	0.1075	0.1954	0.1378	**11**
	300235	1.0000	0.1989	0.1932	0.1577	0.1441	0.0412	0.0386	0.2534	**8**
	300315	0.0510	0.0335	0.0358	0.0319	0.0359	0.0953	0.1244	0.0583	**16**
	002400	0.2616	0.3341	0.4349	0.6042	0.7670	0.0598	0.0468	0.3583	**5**
	**Mean**	**0.2624**	**0.1351**	**0.1559**	**0.2250**	**0.1981**	**0.0691**	**0.0929**	**0.1627**	–
Cultural publishing media enterprise	600037	0.0040	0.0040	0.0050	0.0051	0.0051	0.0049	0.0051	0.0047	**54**
	600088	0.0157	0.0161	0.0118	0.0103	0.0110	0.0126	0.0126	0.0129	**42**
	600373	0.0262	0.0232	0.0204	0.4875	0.0236	0.0197	0.0169	0.0882	**14**
	600551	0.0281	0.0360	0.0383	0.0482	0.0553	0.0327	0.0356	0.0392	**21**
	600757	0.0121	0.0135	0.0136	0.0255	0.0282	0.0258	0.0248	0.0205	**35**
	600825	0.0229	0.0222	0.0224	0.0183	0.0194	0.0194	0.0198	0.0206	**34**
	600831	0.0047	0.0051	0.0058	0.0056	0.0059	0.0067	0.0068	0.0058	**51**
	600880	0.0123	0.0123	0.0133	0.0115	0.0109	0.0152	0.0138	0.0128	**44**
	601098	0.0078	0.0154	0.0159	0.0147	0.0167	0.0153	0.0143	0.0143	**39**
	601801	0.0228	0.0252	0.0276	0.0293	0.0389	0.0534	0.0527	0.0357	**23**
	601928	0.0276	0.0249	0.0350	0.0167	0.0170	0.0139	0.0152	0.0215	**30**
	601929	0.0856	0.0140	0.0138	0.0126	0.0108	0.0060	0.0082	0.0216	**29**
	601999	0.0291	0.0306	0.0325	0.0360	0.0150	0.0188	0.0193	0.0259	**26**
	603000	0.0455	0.0442	0.0476	0.0374	0.0366	0.0417	0.0266	0.0399	**20**
	000156	0.0050	0.0044	0.0052	0.0049	0.0051	0.0051	0.0049	0.0049	**53**
	000665	0.0035	0.0031	0.0022	0.0024	0.0023	0.0027	0.0030	0.0028	**56**
	000793	0.0127	0.0109	0.0106	0.0117	0.0122	0.0152	0.0166	0.0128	**43**
	000917	0.0125	0.0137	0.0141	0.0115	0.0117	0.0132	0.0151	0.0131	**40**
	300027	0.0598	0.0850	0.0317	0.0464	0.0316	0.0706	0.0511	0.0537	**17**
	300059	0.0120	0.0570	0.0623	0.0152	0.0049	0.0028	0.0032	0.0225	**28**
	300104	0.0524	0.1735	0.6234	0.7339	0.7618	1.0000	0.0681	0.4876	**3**
	300133	0.1203	0.1109	0.0803	0.1265	0.2557	0.3707	0.4874	0.2217	**9**
	300148	0.1055	0.0972	0.0811	0.0658	0.0498	0.0545	0.0511	0.0722	**15**
	300226	0.3021	0.4488	0.0360	0.6396	0.9360	1.0000	1.0000	0.6232	**1**
	300251	0.2242	0.3311	1.0000	0.2151	0.1474	0.2104	0.1100	0.3197	**6**
	300291	1.0000	0.7441	0.2156	0.3334	0.5827	0.6422	0.4186	0.5624	**2**
	300336	1.0000	1.0000	0.6647	0.0432	0.0550	0.0768	0.0899	0.4185	**4**
	002095	0.0405	0.0388	0.0391	0.0409	0.0466	0.0423	0.0473	0.0422	**19**
	002181	0.0101	0.0087	0.0083	0.0082	0.0081	0.0090	0.0106	0.0090	**46**
	002238	0.0075	0.0073	0.0076	0.0086	0.0078	0.0069	0.0066	0.0075	**49**
	000681	0.3589	0.2914	0.1565	0.0058	0.2689	0.3001	0.5212	0.2718	**7**
	002071	0.0147	0.0115	0.4514	0.0546	0.0762	0.0422	0.0395	0.0986	**13**
	**Mean**	**0.1152**	**0.1164**	**0.1185**	**0.0977**	**0.1112**	**0.1297**	**0.1005**	**0.1127**	–
Cultural and creative production enterprise	600308	0.0444	0.0424	0.0369	0.0330	0.0309	0.0334	0.0308	0.0360	**22**
	300329	0.0324	0.0194	0.0195	0.0276	0.0202	0.0216	0.0213	0.0231	**27**
	002348	0.0332	0.0381	0.0405	0.0386	0.0125	0.0115	0.0141	0.0269	**25**
	002575	0.0256	0.0271	0.0153	0.0187	0.0136	0.3445	1.0000	0.2064	**10**
	002351	0.0185	0.0177	0.0180	0.0173	0.0155	0.0126	0.0131	0.0161	**38**
	002605	0.0213	0.0206	0.0229	0.0261	0.0209	0.0231	0.0139	0.0213	**31**
	002678	0.0164	0.0118	0.0121	0.0129	0.0129	0.0081	0.0075	0.0117	**45**
	002699	0.0302	0.0194	0.0186	0.0141	0.0169	0.0215	0.0194	0.0200	**36**
	**Mean**	**0.0278**	**0.0246**	**0.0230**	**0.0235**	**0.0179**	**0.0596**	**0.1400**	**0.0452**	–
Cultural leisure and entertainment enterprise	000430	0.0342	0.0177	0.0094	0.0085	0.0072	0.0073	0.0070	0.0130	**41**
	000888	0.0074	0.0060	0.0080	0.0046	0.0045	0.0043	0.0043	0.0056	**52**
	000978	0.0049	0.0011	0.0063	0.0042	0.0042	0.0043	0.0043	0.0042	**55**
	002033	0.0069	0.0058	0.0056	0.0058	0.0059	0.0057	0.0061	0.0060	**50**
	300144	0.0184	0.0170	0.0144	0.0186	0.0213	0.0276	0.0295	0.0210	**33**
	600593	0.0555	0.0135	0.0138	0.0140	0.0166	0.0169	0.0172	0.0211	**32**
	600637	0.0206	0.0251	0.0228	0.0481	0.0349	0.0282	0.0196	0.0285	**24**
	600054	0.0108	0.0068	0.0071	0.0072	0.0067	0.0069	0.0076	0.0076	**48**
	600706	0.0112	0.0070	0.0074	0.0067	0.0071	0.0073	0.0080	0.0078	**47**
	600749	0.0219	0.0241	0.0105	0.0098	0.0147	0.0131	0.0242	0.0169	**37**
	**Mean**	**0.0192**	**0.0124**	**0.0105**	**0.0127**	**0.0123**	**0.0122**	**0.0128**	**0.0132**	–

*The bolded values are the average means for the enterprises.*

Also, Figure 1 demonstrates the top four enterprises’ adjustment and the cultural creative industry’s overall efficiency value.

### Regression Results for Stage Two Stochastic Frontier Analysis Model

In this study, the target value of each DMU obtained in stage one is subtracted from the original input. The capital input slack variable and the labor input slack variable were also determined by applying the two-stage SFA model. It is found that except for a small number of DMUs, when the target value of the capital investment and labor input target value is 0, most DMUs have certain inefficiencies, indicating that Chinese cultural and creative enterprises’ resources are not fully utilized. Therefore, the capital input slack variable and the labor input slack variable were used as the explanatory variables for the SFA regression. The external environmental variables (enterprise size, shareholding structure, talent factor, and government factor) that influence the T.E. of the creative cultural enterprise are used as explanatory variables. The FRONTIER 4.1 software is used to conduct two-stage SFA regression analyses to explore the impact of environmental elements on capital investment and labor input. The analyzed results are displayed in Table 6.

**TABLE 6:** Stochastic frontier analysis regression results.

	Capital investment slack		Labor input slack value	
	Coefficient value	*T*-test value	Coefficient value	*T*-test value
Constant term	7.49	4.34***	4.55	3.05***
Enterprise size (science and technology)	–0.93	−4.27***	–0.51	−2.00**
Ownership structure	–0.36	–0.45	–1.51	−1.81*
Talent factor	–0.74	−3.74***	–0.25	–1.37
Government factor	0.46	4.62***	0.43	4.82***
σ^2^	12.10	6.08***	17.72	3.79***
γ	0.33	2.62***	0.70	7.43***
Log value	−986.103		−933.58	
LR unilateral error test	12.78***		41.09***	

*, **, *** represents 10%, 5%, 1% significance level, respectively.

As indicated in Table 6, although some environmental variables and the regression coefficients of the slack values are not significant, the L.R. unilateral error test passes the test level of 1%. Thus, all environmental variables are still included in the analysis. Further analysis of the repercussion of each environmental variable on the two input slack variables is conducted.

### Enterprise Size (Science and Technology)

The coefficient of SFA regression for the slack value of the two input factors is negative. Thus, an increase in enterprise size (science and technology) can decrease the value of investment slack. Hence, an improvement was observed in the T.E. of the enterprise based on the 1% and 5% significance levels test. Thus, suggesting that the enlargement of the enterprise size (science and technology) will lead to the reduction of the slack input value, thereby improving enterprise T.E. This is consistent with theoretical expectations were the enlargement of enterprises and the advancement of technology, cultural and creative enterprises usually choose to increase capital and labor input to increase creative output and create more value for enterprises.

### Ownership Structure

The SFA regression coefficient of the two input factors’ concentration is positive but did not pass the significance test. Despite this, there still exists some effects. Thus, when the concentration of equity is too high, it will increase the value of investment slack, which is not favorable to improving the T.E. of the listed cultural and creative enterprises.

### Talent Factor

Stochastic frontier analysis regression’s coefficient for the slack value of two input factors is negative and at a 1% significant level, which is the most effective of the four environmental variables. This means that creative talents inhabit an imperative position in the enterprise, and the increase of creative skills can significantly reduce the waste in investment. This is because the region’s economic development in which the company is located can provide a good foundation for introducing high-quality creative talents for cultural and creative enterprises. In addition, economically developed regions attract talents and provide comprehensive supporting services and infrastructure for enterprise development.

### Government Factors

In this manuscript, the government factor values recorded are significant for input inefficiency in the regression model. However, this conclusion does not fully satisfy the theoretical expectation conditions. Notwithstanding, the final results will not be affected since the coefficient value is small, and the significance of the regression is not affected. The result may be due to the perverse selection of variables. The scope of “non-operating income” covers a wide range, including not only government financial support but also education surcharges, fine income, and gains from donations.

### Empirical Results of Investment Adjustment in Stage Three

This study estimated the adjusted capital input and labor input data. The study also used MaxDEA software to analyze the T.E. value of the listed enterprises, which excludes the environmental variables and the random factors. The estimated results are displayed in Table 7.

**TABLE 7:** Technical efficiency values of culture creative enterprise in stage two.

Years		2012	2013	2014	2015	2016	2017	2018	Mean	Ranking

Enterprise										
Creative design service enterprise	300052	0.0504	0.0566	0.0525	0.0912	0.1440	0.0283	0.0341	0.0653	**17**
	300264	0.1386	0.1338	0.1470	0.1634	0.1430	0.1658	0.1908	0.1546	**11**
	300182	0.0849	0.0810	0.1241	0.3708	0.0230	0.1346	0.1978	0.1452	**12**
	300235	1.0000	0.1990	0.1933	0.1577	0.1501	0.0412	0.0386	0.2543	**8**
	300315	0.0510	0.0335	0.0360	0.0359	0.0498	0.1341	0.1700	0.0729	**16**
	002400	0.3021	0.3766	0.4834	0.6471	0.8143	0.0752	0.0563	0.3936	**6**
	**Mean**	**0.2712**	**0.1467**	**0.1727**	**0.2443**	**0.2207**	**0.0966**	**0.01146**	**0.1662**	–
Cultural publishing media enterprise	600037	0.0044	0.0043	0.0054	0.0056	0.0056	0.0054	0.0057	0.0052	**54**
	600088	0.0184	0.0187	0.0120	0.0103	0.0110	0.0127	0.0127	0.0137	**42**
	600373	0.0288	0.0259	0.0220	0.5177	0.0248	0.0206	0.0179	0.0940	**14**
	600551	0.0314	0.0402	0.0429	0.0532	0.0607	0.0408	0.0432	0.0446	**19**
	600757	0.0133	0.0146	0.0145	0.0257	0.0296	0.0273	0.0264	0.0216	**32**
	600825	0.0261	0.0262	0.0267	0.0190	0.0198	0.0195	0.0200	0.0225	**30**
	600831	0.0051	0.0056	0.0064	0.0062	0.0064	0.0072	0.0072	0.0063	**50**
	600880	0.0144	0.0132	0.0142	0.0116	0.0109	0.0153	0.0139	0.0134	**43**
	601098	0.0079	0.0160	0.0171	0.0157	0.0177	0.0163	0.0153	0.0151	**39**
	601801	0.0263	0.0264	0.0276	0.0307	0.0424	0.0576	0.0564	0.0382	**22**
	601928	0.0309	0.0277	0.0371	0.0181	0.0181	0.0153	0.0168	0.0234	**28**
	601929	0.0879	0.0144	0.0142	0.0130	0.0113	0.0063	0.0085	0.0222	**31**
	601999	0.0293	0.0308	0.0327	0.0363	0.0177	0.0196	0.0200	0.0266	**27**
	603000	0.0455	0.0444	0.0504	0.0377	0.0369	0.0420	0.0379	0.0421	**21**
	000156	0.0059	0.0051	0.0061	0.0061	0.0063	0.0062	0.0060	0.0060	**53**
	000665	0.0040	0.0034	0.0026	0.0030	0.0028	0.0031	0.0034	0.0032	**56**
	000793	0.0160	0.0128	0.0116	0.0142	0.0153	0.0153	0.0166	0.0145	**40**
	000917	0.0132	0.0144	0.0149	0.0122	0.0124	0.0140	0.0161	0.0139	**41**
	300027	0.0632	0.0891	0.0376	0.0561	0.0348	0.0744	0.0541	0.0585	**18**
	300059	0.0493	0.0570	0.0624	0.0190	0.0053	0.0029	0.0032	0.0284	**25**
	300104	0.0568	0.1824	0.6493	0.7635	0.8271	1.0000	0.0704	0.5071	**4**
	300133	0.1207	0.1118	0.1106	0.1610	0.2971	0.4212	0.5471	0.2528	**9**
	300148	0.1056	0.0973	0.0812	0.0660	0.0560	0.0686	0.0735	0.0783	**15**
	300226	0.3069	0.4488	0.0372	0.6821	1.0000	0.9088	1.0000	0.6263	**2**
	300251	0.2254	0.3520	1.0000	0.2167	0.2087	0.2121	0.1223	0.3339	**7**
	300291	1.0000	0.7441	0.4115	0.4610	0.7464	0.8486	0.8695	0.7259	**1**
	300336	1.0000	1.0000	0.6627	0.0482	0.0636	0.0903	0.0959	0.4230	**5**
	002095	0.0405	0.0394	0.0396	0.0414	0.0466	0.0423	0.0474	0.0425	**20**
	002181	0.0112	0.0089	0.0084	0.0083	0.0082	0.0090	0.0158	0.0100	**46**
	002238	0.0075	0.0073	0.0079	0.0098	0.0085	0.0079	0.0070	0.0080	**49**
	000681	0.9745	0.9030	0.3175	0.0058	0.5206	0.5558	0.8920	0.5956	**3**
	002071	0.0147	0.0115	0.5015	0.0624	0.0844	0.0450	0.0427	0.1089	**13**
	**Mean**	**0.1370**	**0.1374**	**0.1339**	**0.1074**	**0.1330**	**0.1447**	**0.1308**	**0.1320**	–
Cultural and creative production enterprise	600308	0.0457	0.0437	0.0383	0.0345	0.0325	0.0353	0.0326	0.0375	**23**
	300329	0.0324	0.0195	0.0195	0.0293	0.0202	0.0216	0.0214	0.0234	**29**
	002348	0.0332	0.0385	0.0405	0.0386	0.0125	0.0118	0.0147	0.0271	**26**
	002575	0.0267	0.0281	0.0153	0.0187	0.0136	0.6846	0.4263	0.1733	**10**
	002351	0.0185	0.0178	0.0180	0.0174	0.0155	0.0127	0.0132	0.0162	**38**
	002605	0.0214	0.0207	0.0230	0.0262	0.0210	0.0232	0.0140	0.0214	**34**
	002678	0.0165	0.0127	0.0128	0.0154	0.0153	0.0081	0.0092	0.0129	**45**
	002699	0.0303	0.0194	0.0186	0.0141	0.0182	0.0244	0.0207	0.0208	**36**
	**Mean**	**0.0281**	**0.0250**	**0.0233**	**0.0243**	**0.0186**	**0.1027**	**0.0690**	**0.0416**	–
Cultural leisure and entertainment enterprise	000430	0.0345	0.0177	0.0095	0.0085	0.0072	0.0073	0.0070	0.0131	**44**
	000888	0.0080	0.0067	0.0085	0.0050	0.0047	0.0050	0.0048	0.0061	**51**
	000978	0.0051	0.0027	0.0071	0.0042	0.0042	0.0043	0.0043	0.0046	**55**
	002033	0.0070	0.0060	0.0059	0.0060	0.0059	0.0058	0.0061	0.0061	**52**
	300144	0.0184	0.0170	0.0144	0.0192	0.0224	0.0290	0.0309	0.0216	**33**
	600593	0.0555	0.0135	0.0138	0.0140	0.0166	0.0169	0.0172	0.0211	**35**
	600637	0.0208	0.0265	0.0255	0.0513	0.0378	0.0313	0.0240	0.0310	**24**
	600054	0.0116	0.0073	0.0077	0.0079	0.0074	0.0076	0.0083	0.0083	**48**
	600706	0.0126	0.0074	0.0083	0.0076	0.0078	0.0078	0.0088	0.0086	**47**
	600749	0.0220	0.0241	0.0105	0.0098	0.0147	0.0131	0.0242	0.0169	**37**
	**Mean**	**0.0195**	**0.0129**	**0.0111**	**0.0133**	**0.0129**	**0.0128**	**0.0135**	**0.0137**	–

*The bolded values are the average means for the enterprises.*

**FIGURE 2:**
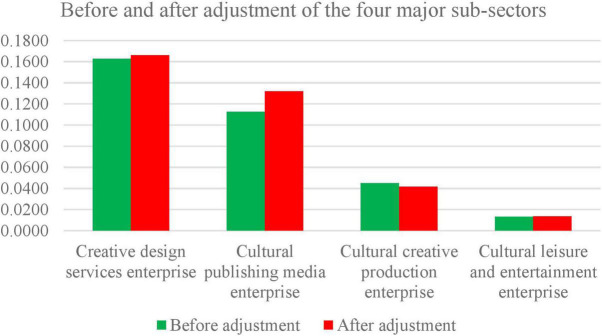
Comparison chart of the four major sub-sectors (before and after the adjustment).

Results from Tables 5, 7 suggest that most companies’ ranking has changed slightly, floating within three enterprises. This shows that environmental variables impact technical efficiency, although it might not be considered the core determinant. The level of T.E. of an enterprise mainly depends on its strength. From the four main sub-sectors as depicted in Figure 2, the creative design service industry, cultural publishing media, cultural leisure, and entertainment industry experienced an upward trend in T.E. The differences in environmental elements usually place these industries in a dire position.

Simultaneously, the technical efficiency value of the cultural and creative industry dwindled based on environmental factors. Its technical efficiency is, however, expected to be improved. Subsequently, after filtering out the brunt of environmental elements, the T.E. value indicated a downward trend. Hence, the disparities in environmental factors may prove advantageous, demonstrating that the current market environment is conducive to developing cultural and creative production enterprises. In summary, these four sub-sectors are all affected by environmental factors. Therefore, the efficiency value obtained by filtering out environmental factors more objectively reflects the enterprises’ actual condition, as shown in Table 7.

Table 7 depicts the differences in T.E. among the various listed cultural and creative enterprises. According to the DEA model, an efficient value is often considered one unless an Inverse-DEA is used; however, most of the 56 enterprises’ efficiency values were less than 0.5, indicating inefficiencies. Therefore, the overall efficiency level of the CCIs is low, with most enterprises showing a dwindling growth in the trend of technological efficiency. Figure 2 below represents the comparison of the four significant sub-sectors adjustments results (before and after adjustments).

The adjustment analysis is performed independently from comprehensive T.E., PTE, and S.E., as shown in Table 8. In general, after adjusting for environmental variables, the efficiency values of various years increased by different amplitudes. Among the enterprises, the scale efficiency increased by 0.0252. From the T.E. results, the adjusted efficiency value indicated fluctuations from 0.0573 in 2012 to 0.0655 in 2018. From the PTE results, the adjusted efficiency value experienced dynamic fluctuations. The efficiency value of PTE in 2018 was lower compared to 2012. Also, the adjusted efficiency value first experienced a decrease in S.E.’s and then increased, reaching the maximum value in 2017 (0.7756). As a result, the efficiency value in 2018 (0.7641) was higher than in 2012 (0.7365). When comparing T.E. to PTE after adjustment, the growth rate of scale efficiency was more significant, suggesting that, provided the same climate and opportunities, the disparity between most cultural and creative listed companies’ current production scale and the ideal production scale is narrowed, indicating an improved scale efficiency. From the above analysis, it can be observed that there were differences after removing the external environment and random factors, the T.E. value of the CCIs changed, and the T.E. values before the adjustment also improved as observed in the adjusted T.E. values in Table 8.

**TABLE 8:** Change of technical efficiency value after adjustment in 2012–2018.

		2012	2013	2014	2015	2016	2017	2018	Mean
**TE**	Before adjustment	0.0537	0.0576	0.0631	0.0537	0.0678	0.0676	0.0669	**0.0615**
	After adjustment	0.0573	0.0607	0.0649	0.0581	0.0728	0.0692	0.0655	**0.0641**
**PTE**	Before adjustment	0.1013	0.0867	0.0896	0.0856	0.0895	0.0922	0.0897	**0.0907**
	After adjustment	0.1149	0.1001	0.1003	0.0934	0.1046	0.1100	0.0993	**0.1032**
**SE**	Before adjustment	0.7016	0.6988	0.7403	0.7287	0.7576	0.7500	0.7608	**0.7340**
	After adjustment	0.7365	0.7340	0.7645	0.7644	0.7753	0.7756	0.7641	**0.7592**

*The bolded values are the average means for the enterprises.*

Table 8 demonstrates the differences in T.E. among the various listed cultural and creative enterprises. For example, the average efficiency value of Hubei Radio and T.V. is the smallest (0.0032), while the average efficiency value of Provincial Radio and Television shares is the highest (0.7259); and, only 4 out of 56 enterprises have an efficiency value above 0.5. Thus, it can be observed that the overall efficiency level of the cultural and creative industries is low. At the same time, most companies recorded negative growth concerning technological efficiency dynamics.

The four significant T.E. values of the high-level industry are: creative design services enterprises, cultural publishing media enterprises, cultural creative production enterprises, and cultural leisure and entertainment enterprises (see Figure 2). Table 9 demonstrates the distribution of enterprise rankings in each sector.

**TABLE 9:** Distribution of enterprises in the four major sub-sectors.

Ranking	Creative design service enterprise	Cultural publishing media enterprise	Cultural and creative production enterprise	Cultural leisure and entertainment enterprise
1–8	33.33%	18.75%	0%	0%
9–16	50.00%	12.50%	12.50%	0%
17–24	16.67%	15.63%	12.50%	10.00%
25–32	0%	18.75%	25.00%	0%
33–40	0%	6.25%	37.50%	30.00%
41–48	0%	12.50%	12.50%	30.00%
49–56	0%	15.63%	0%	30.00%
Total	100%	100%	100%	100%

## Discussion

In the creative design service sector, the company’s efficiency rankings are concentrated in the top 24. The first provincial-owned shares belong to the advertising design industry, with the topmost output capacity. Other companies in this sector are engaged in game software development, audio and video technology software development. The average overall efficiency value of the enterprise is above the industry’s average. Although the cultural creative design service enterprise has developed well in recent years, it is still in the early stages of growth. It faces internal problems such as backward design and transformation technology, communication difficulties for industrial links, shortage of industrial talents, insufficient comprehensive management strength, an immature market, unstable demand, and an imperfect industrial chain. Due to these constraints, the T.E. value of creative service design enterprises began to show a descending trend after reaching the uppermost value (0.2712) in 2012. Therefore, they break the bottleneck and prosper the industry by perfecting the external environment and solving internal problems.

In the cultural publishing media sector, the ranking of enterprise efficiency is more evenly distributed. The top eight and the last eight account for 18.75% and 15.63%, respectively, indicating discrepancies between enterprises’ T.E. values. Among the listed enterprises, the number of cultural publishing media companies is the largest. It comprises 32 enterprises. Many listed enterprises that had low values, such as Xinhua Media (0.0225), Huashu Media (0.006), and Guangdong Media (0.01), transformed from conventional enterprises, with their assets turning into losses after the restructuring. The overall productivity of these companies is low. None of them reached 0.1. On the other hand, the efficiency values of Internet service companies were observed to be generally high, such as Eastern Fortune (0.7259), LeTV (0.5071), and Shanghai Steel Union (0.6263). Therefore, to successfully leap from traditional industries to the CCIs, it is inevitable to constantly adjust the economic structure, enhance low-end industries through creativity, and further upgrade the cultural sector from primary form to network, digital, and cultural integration.

Among the cultural and creative production sectors, only 12.5% of the companies appeared in the top 16. Huatai Co., Ltd., which engrosses mainly in cultural paper manufacturing, is the most advanced. The least developed industry is the Meisheng Culture, a cross-disciplinary, cross-industry, cross-platform eco-culture enterprise that builds a leading global intellectual property ecosystem. As an outcome of the enterprise’s strategic transformation recently reported, the company has heavily invested in an extensive layout system. Hence, many investment acquisitions and mergers do not reflect its advantages, but its prospects are projected to increase.

The ranking is akin to the cultural leisure, entertainment, and creative production sectors, concentrated in destinations with lower overall efficiency value among the four sub-sectors. This phenomenon reflects the absence of efficiency in China’s management and development of tourism resources. It is still unable to explore the cultural and creative elements effectively. Thus, despite China’s vast land and abundant cultural resources, the lack of creativity has stagnated the tourism and leisure industry.

## Conclusion

This research applied the three-stage DEA and modified SFA model to measure the technical efficiency (T.E.) of 56 listed cultural and creative enterprises from 2012 to 2018. The results disclosed some differences among the T.E. values of the 56 enterprises in stage one and stage three, but it was not significant to affect the outcome of this study. The disparities in CCIs of the enterprises are linked to the early stage of CCIs development in China with a low aggregate efficiency value. The differences show that the T.E. of creative cultural enterprises is significantly affected by environmental elements and white noise. Thus, there is a greater need for collaboration, support, and enhancement in the activities of the CCIs to serve as an engine for industrial and economic growth. The analysis above shows that the overall value of T.E. of the CCIs is generally low. As a strategic emerging industry in the new century, enterprises have not given maximum concentration to their social responsibilities. There are several reasons behind this phenomenon. Such as lack of advanced technology and high-end creative talents and in the early stage of industrial development, a considerable amount of resources is injected, which makes the investment inefficient, resulting in misuse of resources.

### Managerial Implications

Based on the problems enumerated in section 6.1 of this current study, the subsequent suggestions are set forth to make the organizations more sustainable: First, at the micro-level, each company should review the situation and allocate the inputs of different production factors reasonably and effectively, such as increasing R&D expenditures and reducing labor inputs. Secondly, from the stage two analysis of the models used, creative talents occupy a vital position in the enterprise. Enterprises can develop a talent training mechanism that meets the market demand. An incentive and promotion mechanism that attracts creative talents can also be implemented. Such a strategic initiative would reduce investment in inefficiency and increase creative output. Furthermore, talent training should cultivate many knowledge-based skills, an inclusive attitude, and creative ability.

In summary, the cultural and creative enterprises should not be limited to the complete change and advancement of traditional culture but need to open up more business opportunities, adapt to the changing trend of the times, and integrate the internet plus culture with the CCIs (Li, 2020; Bertoni et al., 2021). For example, in China, Palace Museums have received much attention in recent years. With the broadcast of large-scale documentaries such as “I am in the Forbidden City,” the Forbidden City slowly entered people’s lives. The president of the Palace Museum, Shan Yuxiang, said on the premise: “The ancient buildings and cultural relics have a splendid past, and they should have a dignified present and should also healthily move toward the future.” Thus, greater emphasis has been placed on studying cultural relics. The Forbidden City’s official website has been updated regularly, and an official microblog has also been launched to promote cultural relics. Also, the Forbidden City’s archives have been shown online, and cultural and creative products to “experience” the ancient buildings and cultural relics have been launched, embodying a superb standard of internet high technology and culture convergence. These initiatives have created an excellent connection and knowledge about the Forbidden City and have contributed annual sales of over one billion yuan of innovative products.

At the macro level, the development of the CCIs should incorporate the historical opportunities of the “Belt and Road Initiative” and the Yangtze River Economic Belt strategy implemented by the government. The development of global CCIs has gradually shown the trend of aggregation, scale, and regional distribution. Their growth is not balanced in all regions but concentrated in robust scientific and technological strength, a developed market economy, and rich cultural resources. China must also have a strong CCI power belt and take advantage of the Yangtze River economic belt resources led by Shanghai to bolster other regions’ harmonized development to build a world cultural power. Besides, the government should also concentrate on improving the environment for the survival and growth of cultural and creative enterprises. To effectively resolve the imbalance in the distribution of talents, there should be an excellent statistical survey of skills in various regions, compile and publish a catalog of talent shortfalls within the areas, and highlight the job requirements and government support policies.

Finally, there should be a framework to guide and improve the financing channels of the CCIs, regulate and manage the financing activities of various regions, and help some small and micro-enterprises that are just beginning to resolve the pertinent problem of financing difficulties gradually.

### Theoretical Contribution

The analysis and review conducted by the authors of this present study revealed that most researchers of the existing literature mostly used the traditional DEA model and SFA model, which have certain shortcomings. This current study contributes to the existing knowledge by adopting a three-stage DEA model to measure the technical efficiency of cultural and creative enterprises, taking the influence of environmental and random factors on the assessment into account. The improved models adopted effectively solve some problems caused by missing data and reduce errors, making the measurement data more consistent with the real level of enterprise operations.

### Practical Implication

Developing a creative economy is an established national policy for sustainable development in developed countries, and an essential guide for comprehensive development in developing and underdeveloped countries. The social benefits of cultural and creative industries have increased significantly, directly satisfying the public’s unique needs, growing faster, and highlighting the need for balanced CCIs. New industries and models, led by the Internet, drive cultural and creative industry development. Therefore, this study adopts a three-stage DEA model to measure the technical efficiency value of China’s listed cultural and creative enterprises. The approach adopted is necessary to fully understand the industry’s development status, measure the technical efficiency of cultural and creative enterprises, and explore the influencing factors in the evolution process.

### Limitations and Future Research

The cultural and creative industry is a strategic emerging industry to aid development in China; however, its data gathering and documentation started late, so there are fewer data and statistical reports available in this industry. Due to the data unavailability, the period for the statistical was restricted to 2012 through 2018. As data are being made accessible in the recent period, the authors planned to analyze technical efficiency changes in the CCIs from a long-term perspective in the future. Furthermore, the current development of cultural and creative industries has attracted the integration of industries and as a driving factor for other industrial development; however, the existing literature is limited in this field; hence, further studies would be conducted in our subsequent studies.

## Data Availability Statement

The raw data supporting the conclusions of this article will be made available by the authors, without undue reservation.

## Author Contributions

ML conceptualized the study. HS designed the study. FA wrote and reviewed the manuscript. JS contributed to the acquisition of data. WH analyzed and interpreted the data. All authors contributed to the article and approved the submitted version.

## Conflict of Interest

The authors declare that the research was conducted in the absence of any commercial or financial relationships that could be construed as a potential conflict of interest.

## Publisher’s Note

All claims expressed in this article are solely those of the authors and do not necessarily represent those of their affiliated organizations, or those of the publisher, the editors and the reviewers. Any product that may be evaluated in this article, or claim that may be made by its manufacturer, is not guaranteed or endorsed by the publisher.
